# A bibliometric study for global hotspots and trends in animal-assisted interventions (1983–2023)

**DOI:** 10.3389/fpsyt.2025.1490122

**Published:** 2025-04-16

**Authors:** Xiaowei Feng, Shanguang Zhao, Dong Zhang, Qing Yi, Yanlan Chen, Xinding Zhang

**Affiliations:** ^1^ Faculty of Physical Education/Faculty of Football, Hainan Normal University, Hainan, China; ^2^ Expert Workstation in Sichuan Province, Chengdu Jincheng College, Chengdu, China; ^3^ Department of Sports Studies, Faculty of Educational Studies, Universiti Putra Malaysia, Kuala Lumpur, Malaysia; ^4^ Faculty of Sports and Exercise Science, Universiti Malaya, Kuala Lumpur, Malaysia; ^5^ Faculty of Education, Universiti Malaya, Kuala Lumpur, Malaysia

**Keywords:** animal-assisted interventions, bibliometrics, research trends, nonpharmacological therapies, vulnerable groups

## Abstract

**Background:**

As a therapeutic approach, Animal-Assisted Intervention (AAI) has gained increasing recognition for enhancing both psychological and physical health. However, bibliometric studies in this field remain scarce.

**Methods:**

This study aims to analyze AAI-related research from 1983 to 2023 using bibliometric methods. It examines sources of literature, core journals, highly cited documents, country and institutional distribution, prolific authors, and high-frequency keywords while tracking the evolution of research themes in AAI. A thematic search using the Boolean operator “OR” and AAI-related keywords in the Web of Science database yielded 405 articles, and data mining and visualization of these results were performed.

**Results:**

The findings reveal a substantial increase in the number of published articles and citations over the past decade, indicating a rising research interest in this field. The United States and Purdue University have played a leading role in this area. Currently, AAI research is shifting from basic studies to intervention strategies targeting specific populations and diseases. Future research trends may include enhanced international collaboration, standardization of research methods, and the development of more targeted interventions.

**Conclusion:**

These findings provide researchers, funding agencies, and policymakers with scientific insights and recommendations for future research directions in AAI.

## 1 Introduction

Animal-assisted interventions (AAI) comprise preventive strategies that employ animals to promote overall health and well-being through non-educational, non-pharmacological, and mediatory methods. These interventions include emotional, psychological, and physical interactions among people, animals, and their surroundings (1). Consequently, AAI is frequently employed to address various behavioral, psychological, and emotional issues that individuals may experience (2). Currently, AAI is recognized as either an alternative or a complementary approach to conventional medical or psychological treatments (3, 4), and it can effectively improve emotional health, reduce anxiety, and lower stress levels (5–7).

Since the introduction of the concept of AAI by American psychiatrist Boris Levinson in 1962 (8), interest in the therapeutic benefits of AAI has steadily increased over the past 60 years, leading to its incorporation into various medical and psychological treatment programs. This increasing popularity is reflected in the substantial rise in academic publications exploring various aspects of AAI, including its efficacy, potential mechanisms, and applications across different patient populations. Existing systematic reviews and meta-analyses have provided in-depth analyses and discussions of AAI from different research perspectives, such as for psychiatric patients (9), individuals with intellectual disabilities (10), dementia (11), prison populations (12), and the effectiveness of AAI (13, 14).

Although the benefits of AAI have been widely expounded, its integration into mainstream medical care and treatment practice is not without challenges. This includes ethical issues (15), internal effectiveness (16), and other factors that hinder the wide adoption and scalability of AAI intervention measures. In view of increasing research in this field, it is necessary to systematically evaluate its progress and emphasize emerging trends to guide future progress.

Bibliometric analysis involves the quantitative assessment of academic literature, offering valuable insights into research trends, key thematic areas, and influential contributors within a specific field. Unlike traditional systematic reviews and meta-analyses, bibliometric analysis provides a more systematic and intuitive method for revealing the current state and development of research topics (17).

However, despite the growing literature on AAI, bibliometric studies that comprehensively depict the knowledge landscape in this field are significantly lacking. To address this gap, this paper employs bibliometric methods to analyze the sources, countries, institutions, authors, and thematic evolution within the AAI research domain. This approach not only maps the knowledge landscape of the field but also aids in understanding future research directions and potential areas for interdisciplinary collaboration.

## 2 Materials and methods

### 2.1 Data collection and search strategies

This study utilized the Web of Science Core Collection (WoSCC), a multidisciplinary scientific citation index database that includes the most important international journals. As the research did not involve human or animal subjects and all data used were sourced from public databases, ethical approval was not required.

**Figure 1:**
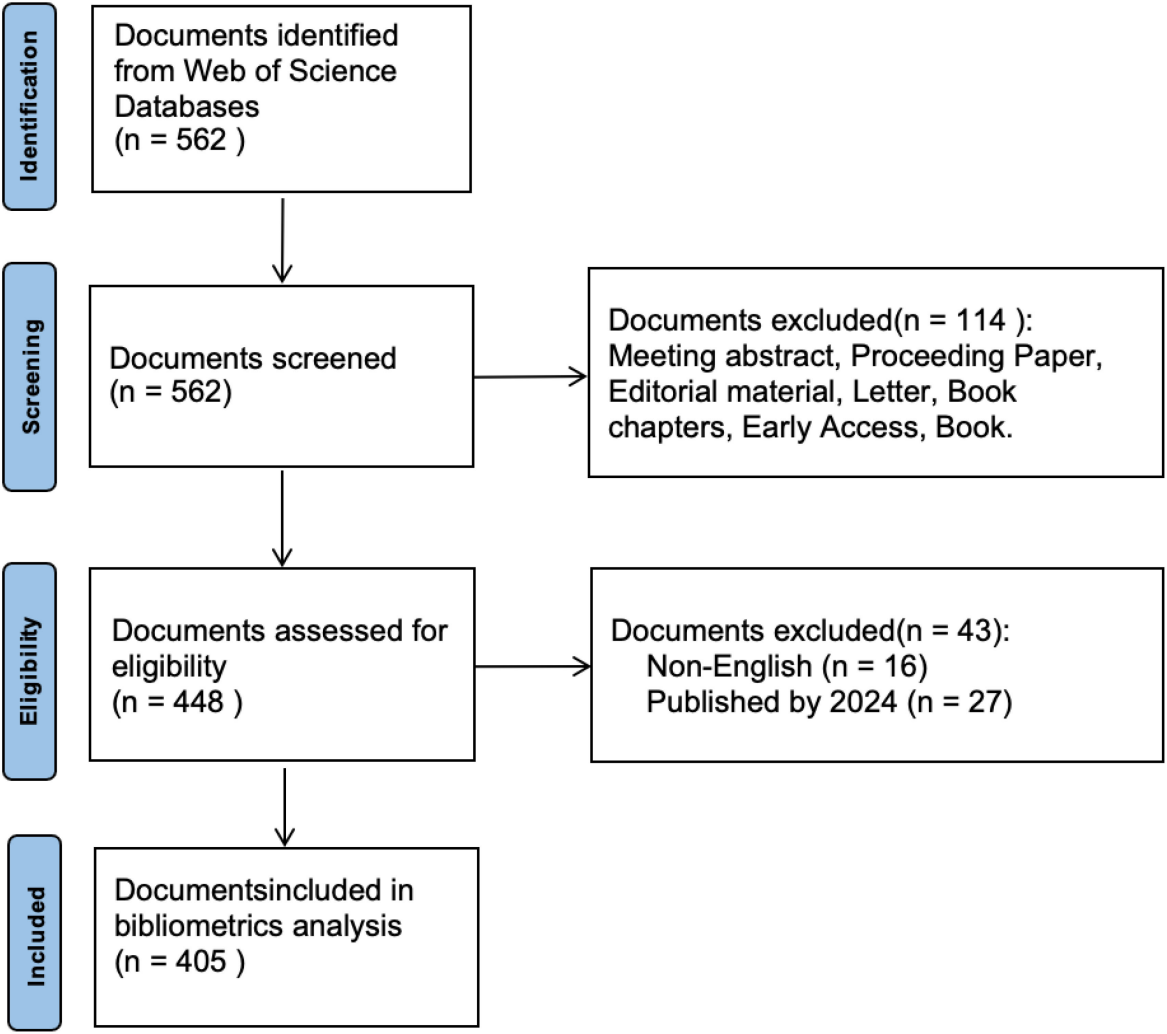
PRISMA flow diagram illustrating the process of document selection.

The study adhered to the PRISMA guidelines (18). We conducted our first search on August 5, 2024, and our last search on August 30. The search strategy involved using the Boolean operator “OR” to combine keywords related to AAI. The keywords were selected based on common terminology and the most widely used terms in the literature on AAI. The search query was as follows: TS = (“animal-assisted intervention” OR “animal-assisted therapy” OR “animal-assisted activity” OR “pet therapy”), and no search date range has been set. This search strategy initially identified 562 records. However, 27 records published in 2024 were excluded due to their ongoing updates. In addition, non-English articles (n=16) were excluded to ensure linguistic consistency and facilitate analysis, as English publications were considered a quasi-restrictive criterion. The decision to limit the search to English articles was made to avoid potential translation bias and the challenge of interpreting foreign language material. To ensure the originality of findings and minimize bias, we included only original research articles (n=320) and review articles (n=85). These articles are classified according to their abstract and content, with original research defined as empirical research presenting new findings and review articles as articles summarizing and synthesizing existing research. Finally, 405 records were included in the bibliometric analysis (see **Figure 1**).

### 2.2 Bibliometric analysis

Bibliometric analysis is a quantitative research method that mainly focuses on academic productivity and uses published scientific literature (research articles, books, conference records, etc.) to measure research activities in specific fields (19). This research method plays a key role in data classification and information acquisition. It can reveal research trends, knowledge structure, and academic influence, thus providing accurate, reliable, and sufficient information support (20).

### 2.3 Data mining and visualization

Descriptive data on authors, countries, institutions, and journals from WoSCC were organized using Microsoft Excel 2019. The Journal Citation Report (JCR) Science Edition (2023) provides relevant data on the journal impact factor (IF), category, and category quartile (CQ). Geographical distribution maps were created using the online visualization tool Datawrapper (https://www.datawrapper.de/tables, accessed August 22, 2024). Finally, the bibliometric analysis tool Biblioshiny was employed to display Bradford’s law model, national collaboration networks, and trends in thematic evolution.

## 3 Results

### 3.1 Analysis of publication and citation trends

**Figure 2:**
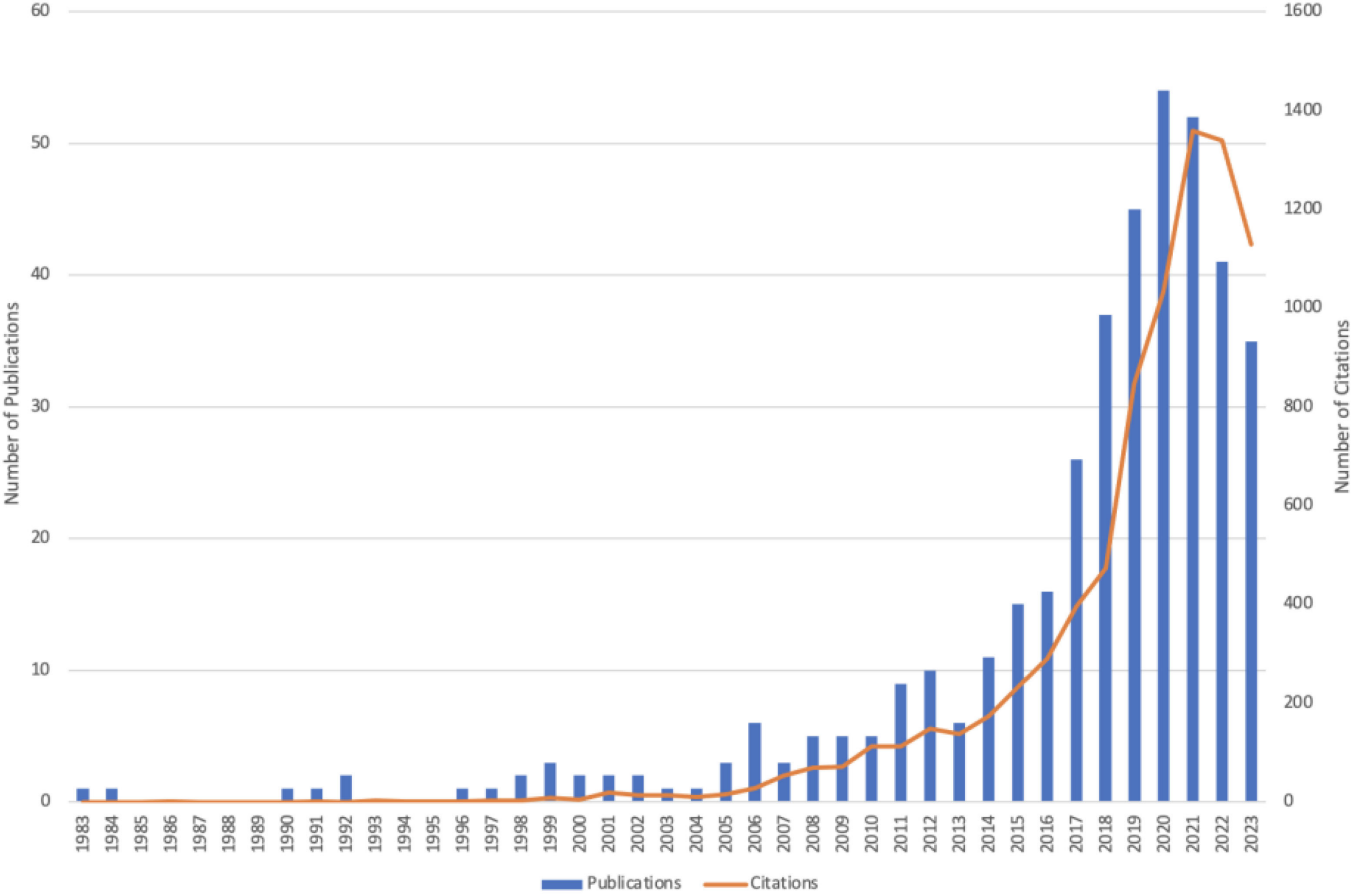
The yearly count of publications and citations from 1983 to 2023.

Based on the search terms and inclusion criteria, we selected and analyzed 405 articles published between 1983 and 2023, with a total of 8,846 citations, averaging 21.84 citations per article and an H-index of 52. As shown in **Figure 2**, the number of articles and citations in this field experienced rapid growth from 2013 to 2023, with 338 articles published during this decade, accounting for 83.5% of the total literature. Notably, the highest number of publications occurred in 2020 (n=54), while citations peaked in 2021 (n=1,360).

### 3.2 Journal source analysis

**Figure 3:**
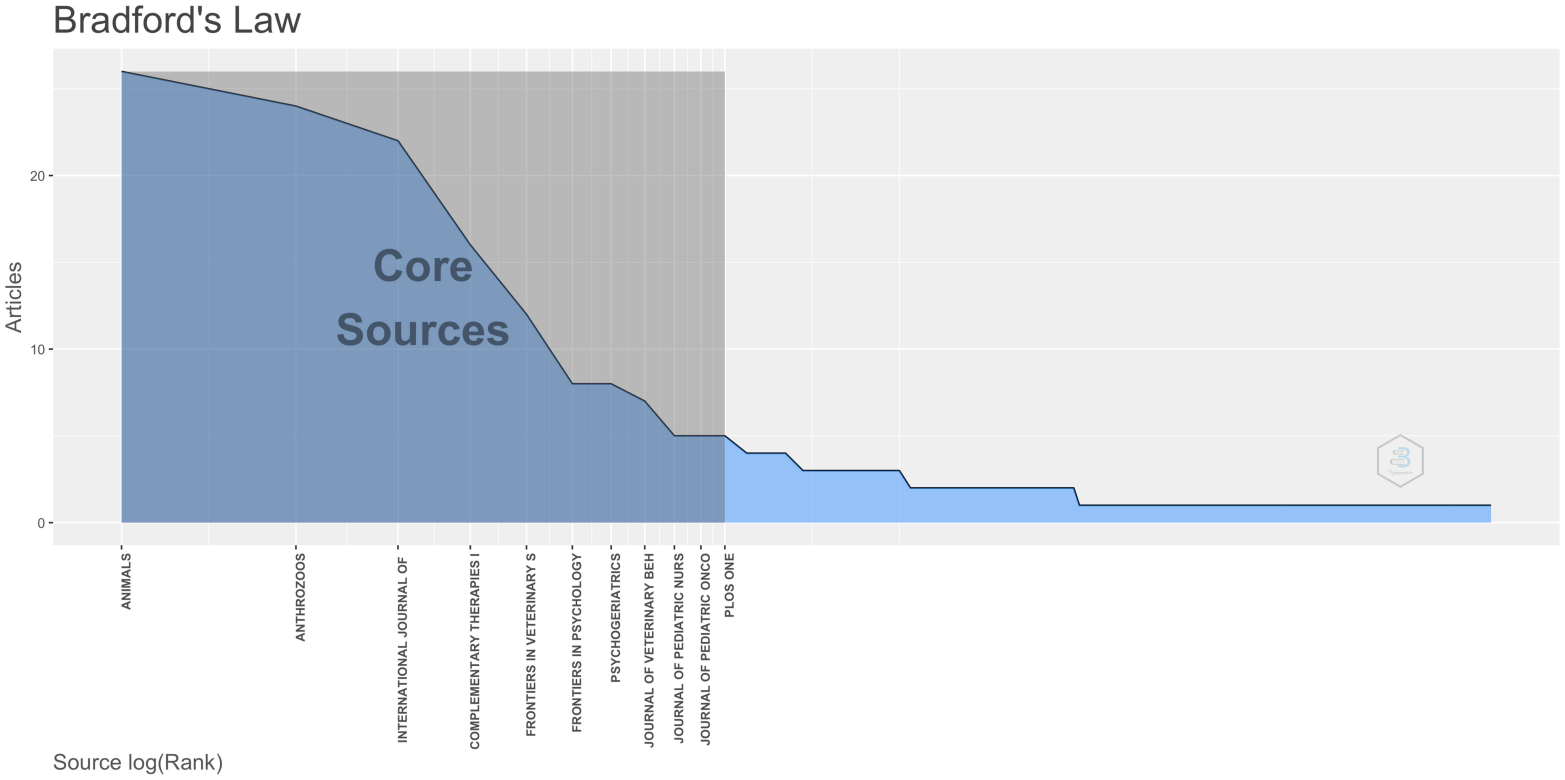
Bradford’s Law presents 11 core journals.

Bradford’s Law, commonly used in bibliometrics to reveal the distribution patterns of scientific literature, indicates that a small number of core journals contain the majority of significant research publications, while a large number of peripheral journals contain fewer relevant articles (21). According to this law, AAI research literature is sourced from 231 scientific journals, with the top 11 core journals being the most prolific contributors to AAI publications (**Figure 3**). These core journals represent 4.8% of the total journal sources (11/231) but account for 138 publications, which constitute 34.1% of the total literature.

To further analyze the characteristics of these journals, we obtained relevant information from the JCR Science Edition (2023) (**Table 1**). In terms of quartiles, there are 3 journals in Q1, 6 in Q2, and 1 in Q3. Among them, *Animals* published the highest number of articles, totaling 26, which represents 6.4% of the total literature (26/405). *PLOS One* has the highest IF at 2.9. The categories of these journals indicate a broad research scope on AAI, encompassing fields from veterinary and environmental sciences to psychology, nursing, and integrative medicine.

**Table 1:** The top 11 productive journals of AAI.

Journals	Articles(%)	IF 2023	CQ 2023	JCR Category
Animals	26 (6.420)	2.7	Q1	Agriculture, Dairy & Animal Science; Veterinary Sciences
Anthrozoos	24 (5.926)	1.7	Q2	Sociology; Veterinary Sciences
International Journal of Environmental Research and Public Health	22 (5.432)	N/A	N/A	Environmental Sciences; Public, Environmental & Occupational Health; Public, Environmental & Occupational Health
Complementary Therapies in Clinical Practice	16 (3.951)	2.2	Q2	Integrative & Complementary Medicine
Frontiers in Veterinary Science	12 (2.963)	2.6	Q1	Veterinary Sciences
Frontiers in Psychology	8 (1.975)	2.6	Q2	Psychology, Multidisciplinary
Psychogeriatrics	8 (1.975)	1.7	Q3	Geriatrics & Gerontology; Psychiatry
Journal of Veterinary Behavior Clinical Applications and Research	7 (1.728)	1.3	Q2	Behavioral Sciences; Veterinary Sciences
Journal of Pediatric Nursing-Nursing Care of Children Families	5 (1.235)	2.1	Q2	Nursing; Pediatrics
Journal of Pediatric Oncology Nursing	5 (1.235)	1.9	Q2	Nursing; Oncology
Plos One	5 (1.235)	2.9	Q1	Multidisciplinary Sciences

IF, Impact Factors; CQ, category Quartile; JCR, Journal Citation Report.

### 3.3 Analysis of highly cited literature

**Table 2** lists the ten most cited studies, which are published in ten different journals, with four of these journals appearing in the first quartile of the JCR. Notably, six of the ten most cited articles originate from the United States. These ten articles focus on various applications and benefits of AAI in fields such as autism, depression, dementia, and trauma. Collectively, they highlight the therapeutic potential of AAI in enhancing human well-being, particularly among vulnerable populations. Each article serves as a cornerstone in its respective field, advancing the understanding and practice of AAI, and contributing to the broader domain of human-animal interaction research.

**Table 2:** The characteristics of the top 10 highly cited documents on the research of AAI.

TC	Article Titles	Journals	Published Years	Countries	IF 2023	CQ 2023
230	Animal-Assisted Intervention for Autism Spectrum Disorder: A Systematic Literature Review (22)	Journal Of Autism and Developmental Disorders	2013	Australia	3.2	Q1
175	Do animal-assisted activities effectively treat depression? A meta-analysis (23)	Anthrozoos	2007	USA	1.7	Q2
170	The Human-Companion Animal Bond: How Humans Benefit (24)	Veterinary Clinics of North America-Small Animal Practice	2009	USA	1.9	Q2
167	Evidence-Based Nonpharmacological Practices to Address Behavioral and Psychological Symptoms of Dementia (25)	Gerontologist	2018	USA	4.6	Q1
161	The Utilization of Robotic Pets in Dementia Care (26)	Journal of Alzheimer’s Disease	2017	USA	3.4	Q2
147	Play and pets: The physical and emotional impact of child-life and pet therapy on hospitalized children (27)	Children’s Health Care	2002	USA	0.7	Q4
133	Animal-assisted therapy for dementia: a review of the literature (11)	International Psychogeriatrics	2006	Australia	4.6	Q1
122	Animal-assisted interventions for elderly patients affected by dementia or psychiatric disorders: A review (28)	Journal of Psychiatric Research	2013	Italy	3.7	Q1
120	The benefits of and barriers to using a social robot PARO in care settings: a scoping review (29)	BMC Geriatrics	2019	Canada	3.4	Q2
110	Animal-Assisted Intervention for trauma: a systematic literature review (30)	Frontiers in Psychology	2015	USA	2.6	Q2

TC, Total Citations; IF, Impact Factors; CQ, Category Quartile.

### 3.4 Analysis of country/region and institutional productivity

**Figure 4:**
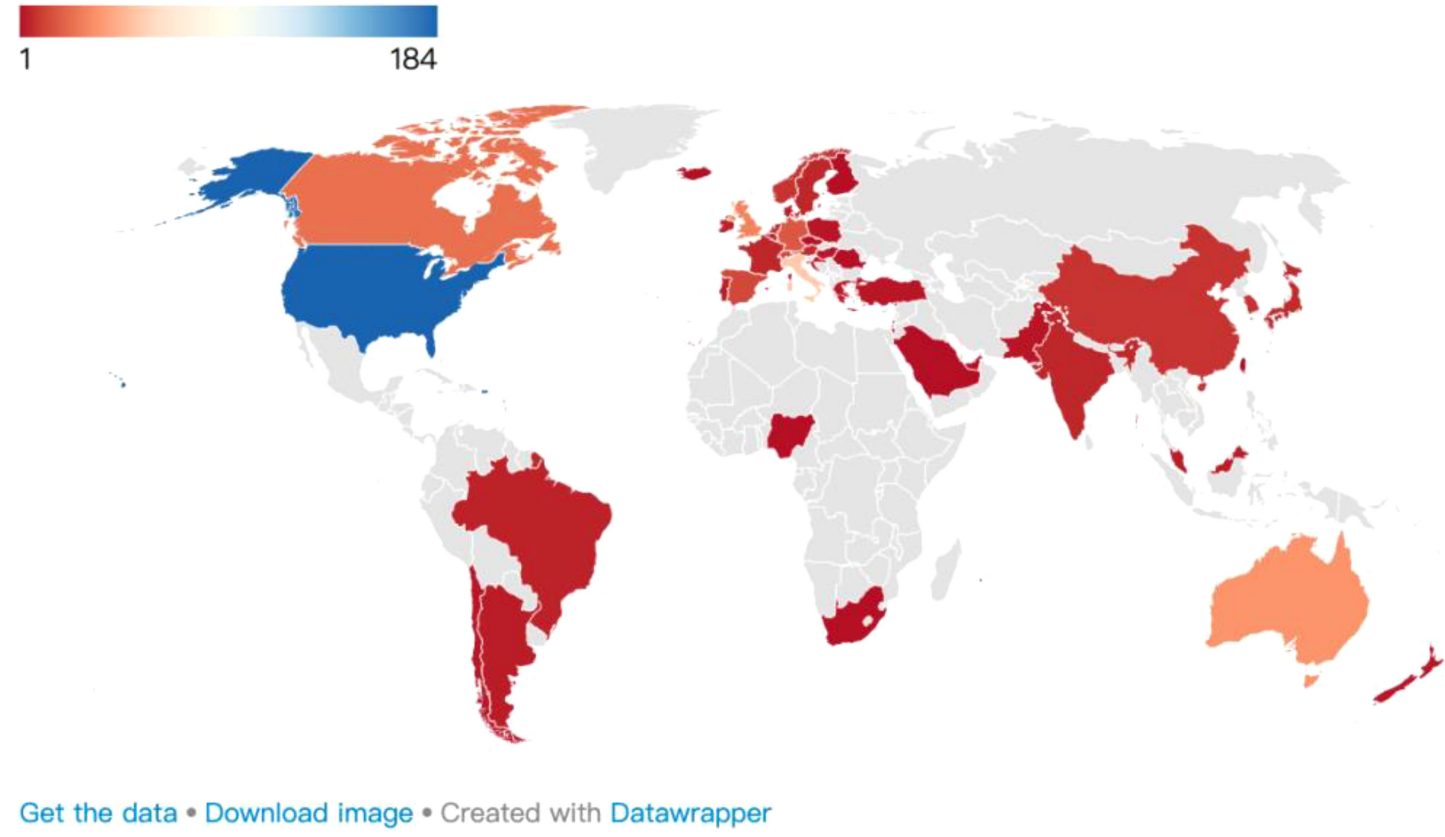
Geographical distribution map of AAI research publications.

Globally, 47 countries/regions have participated in AAI research (**Figure 4**). Regarding research output, the top three countries are the United States (n=184), Italy (n=53), and Australia (n=35).

From an impact perspective, the countries with the highest relevance and total citation (TC) counts include the United States (H=39, TC=4,508), followed by Italy (H=22, TC=1,454) and Australia (H=16, TC=1,086). However, when calculated by citations per article (CPA), the top three countries are Australia (CPA=31.03), Italy (CPA=27.43), and the United States (CPA=24.5) (**Table 3**). The research outputs on AAI predominantly originate from universities worldwide, with Purdue University (n=24) in the United States making the greatest contribution.

**Table 3:** Top 10 countries/regions and institutions with the most research on AAI.

Countries	Articles	TC	H-Index	CPA	Top Country Institutions*	Top Institution Articles (%)
USA	184	4508	39	24.5	Purdue University	24 (13.043%)
Italy	53	1454	22	27.43	University Of Naples Federico II	10 (18.868%)
Australia	35	1086	16	31.03	University of Queensland	9 (25.714%)
United of Kingdom	27	518	12	19.19	Cardiff University Mars State University Of New York Suny System Suny Fredonia University Of London Waltham Petcare Science Institute	3 (11.111%)
Canada	24	564	9	23.5	University of Saskatchewan	6 (25.000%)
Germany	17	199	6	11.71	Helmholtz Association Helmholtz Zentrum Dresden Rossendorf Hzdr Technical University of Munich Technische Universitat Dresden University of Munich	2 (11.765%)
Spain	13	116	5	8.92	Complutense University of Madrid	3 (23.077%)
Switzerland	13	213	7	16.38	University of Basel	9 (69.231%)
Norway	8	187	6	23.38	Norwegian University of Life Sciences University of Oslo	3 (37.500%)
China	8	148	6	18.5	Hefei Normal University Peking University Shenzhen University	2 (25.000%)

TC, Total Citations; CPA, Citations per Article,* Parallel institutions publish the same number.

**Figure 5:**
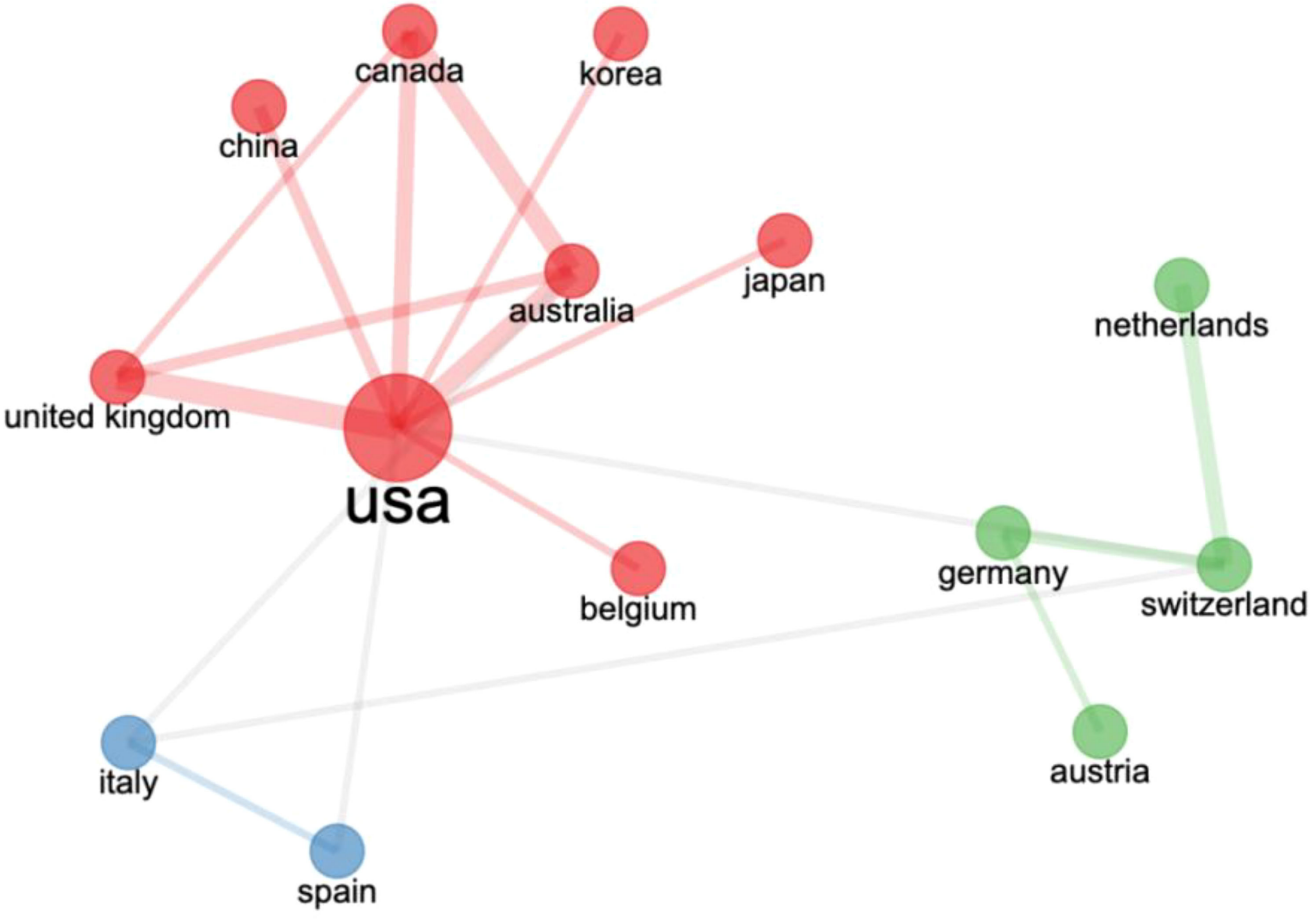
AAI research country collaboration map.

**Figure 5** illustrates the network structure of international collaborations, which is divided into three clusters. The red cluster highlights close collaborative relationships between the United States and China, Canada, South Korea, the United Kingdom, Australia, Japan, and Belgium. The blue cluster includes Spain and Italy, while the green cluster comprises the Netherlands, Switzerland, Germany, and Hungary. These international collaborations have effectively facilitated progress in AAI research globally.

### 3.5 Analysis of leading authors

The top ten authors in terms of research output predominantly come from the United States and Italy (**Table 4**). This finding aligns with the results of the country output analysis and further underscores the research prominence of these two countries in the field of AAI. Scholars from both countries excel in terms of research quantity, quality, and citation counts, thereby consolidating their leading position in global AAI research.

**Table 4:** The leading 10 authors in the field of AAI research.

Authors	Countries	Articles	H-index	TC
O’Haire, Marguerite E	USA	20	15	848
Hediger, Karin	Switzerland	8	5	88
Menna, Lucia Francesca	Italy	8	7	223
Santaniello, Antonio	Italy	8	7	223
Rodriguez, Kerri E	USA	8	6	225
Krause-Parello, Cheryl A	USA	7	6	164
Gee, Nancy R	USA	7	6	195
Friedmann, Erika	USA	6	5	336
Fioretti, Alessandro	Italy	5	5	147
Borgi, Marta	Italy	4	4	161

TC, Total Citations.

### 3.6 Analysis of high-frequency words

**Figure 6:**
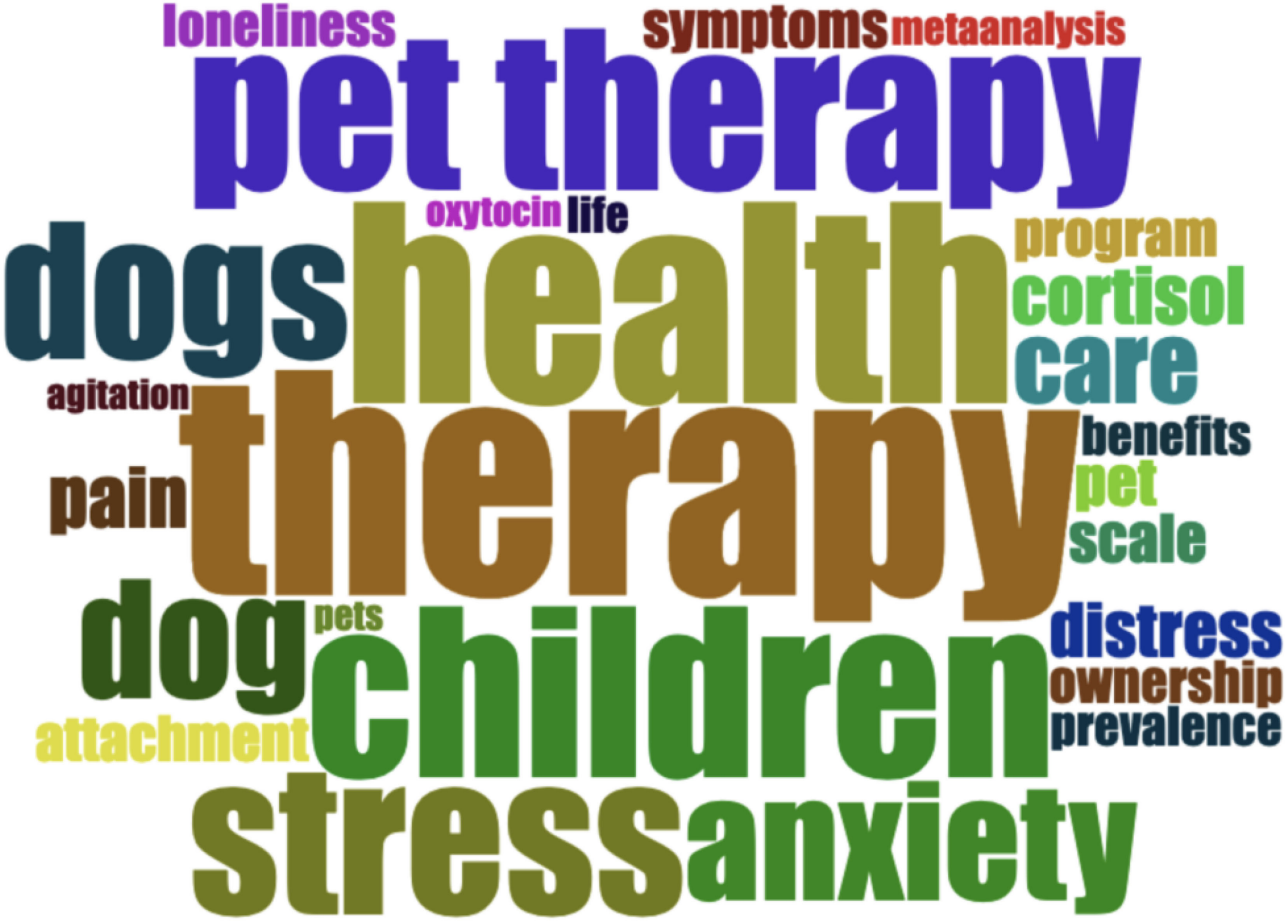
Word cloud map of AAI research.

In order to quickly identify the research topics and hotspots in the AAI field, we use keyword plus to analyze high-frequency keywords because keyword plus can reflect the research trend more accurately than the keywords provided by the author (31). **Figure 6** shows the word cloud map generated by Keyword Plus, in which the ten keywords with the highest frequency are: therapy (74 times), health (66 times), children (56 times), pet therapy (53 times), depression (52 times), stress (50 times), animal-assisted therapy (45 times), dogs (45 times), anxiety (39 times), and dog (39 times).

### 3.7 The theme evolution trend of AAI research

**Figure 7:**
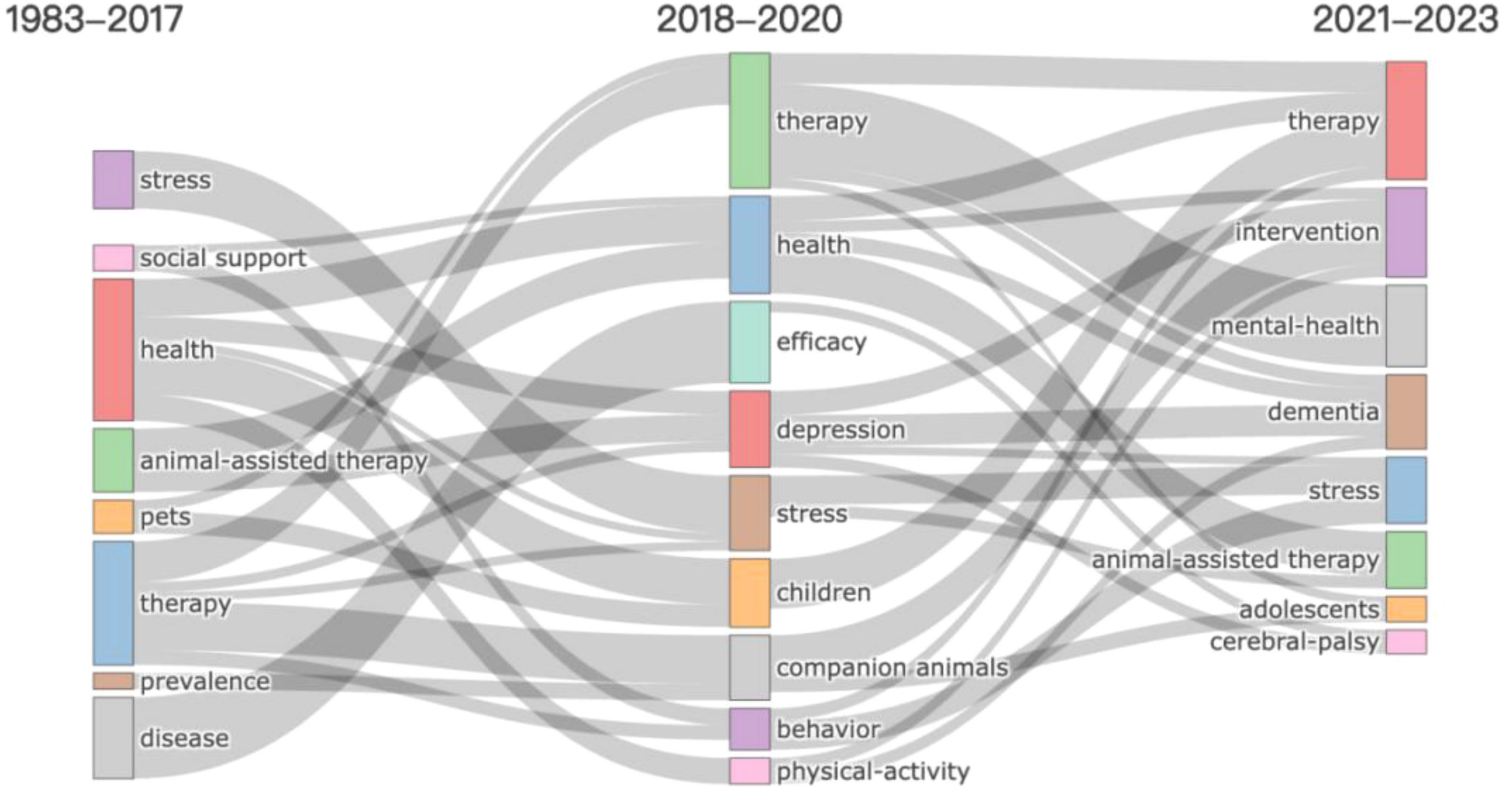
Trend diagram of theme evolution of AAI research.

Over the decades, the focus and terminology of AAI research have undergone significant evolution. By analyzing thematic terms from different periods (1983-2017, 2018-2020, and 2021-2023), the development and changes in the field can be traced, highlighting shifting priorities and emerging trends. We utilized keyword plus to reveal the thematic evolution in AAI research through a Sankey diagram (**Figure 7**), as keyword plus offers a more detailed description of trends compared to author-defined keywords (31), thus facilitating the identification and association of different research areas (32).

#### 3.7.1 Foundational research and broad applications (1983–2017)

From 1983 to 2017, AAI research primarily focused on foundational theories and a wide range of applications. The research emphasized fundamental themes such as stress, social support, health, and the overall efficacy of pet therapy within the context of disease prevalence. During this period, researchers aimed to understand the effects of AAI in various contexts. For instance, theoretical frameworks related to AAI highlighted its impact on psychological health factors, such as stress, as a form of social support (33). Additionally, studies explored the impact of pet therapy on diverse populations, including hospitalized children (27), patients with terminal cancer (34), and elderly individuals with psychiatric disorders (35). These studies not only focused on building theoretical foundations but also explored the potential integration of AAI into practical therapies, assessing its benefits for human health. The research conducted during this period laid the theoretical and empirical groundwork for subsequent stages.

#### 3.7.2 Emphasis on efficacy (2018–2020)

Between 2018 and 2020, the focus of AAI research gradually shifted towards a more refined understanding of efficacy, concentrating on specific therapeutic outcomes and results. Research during this stage began to more precisely evaluate the impact of AAI on particular psychological conditions, such as depression and anxiety and further explored the multidimensional effects of AAI on physical activity and behavior.

Studies from this period demonstrated significant effects of AAI in alleviating depressive symptoms (36, 37), and improving social behavior (38–40), as well as promoting physical activity (37, 41). These findings deepened the understanding of AAI’s comprehensive effects and provided empirical support for its application in various health conditions.

#### 3.7.3 Specialized interventions and targeted populations (2021–2023)

From 2021 to 2023, AAI research has transitioned towards more specialized interventions and applications for specific populations. This phase has focused on the development and implementation of personalized treatment approaches, particularly for populations with special needs. Key themes include interventions for specific conditions such as mental health issues, dementia, adolescents, and cerebral palsy.

Research indicates that AAI, as an adjunctive intervention, has shown positive effects in the treatment of neurodegenerative diseases, including dementia [e.g., (42–45)], and neurodevelopmental disorders, including cerebral palsy [e.g., (46–48)]. These studies not only refine intervention strategies but also provide concrete evidence of AAI’s effectiveness in specific populations. The outcomes of this phase establish a solid foundation for further research into the application and optimization of AAI for special populations, while advancing the development of personalized treatments.

The analysis of the topic evolution is helpful for researchers and practitioners in the field of AAI to sort out the topic focus in different stages of AAI and the trend of future development, so as to provide ideas for finding research directions.

## 4 Discussion

This bibliometric analysis reveals trends and hotspots in AAI research. Since 1983, particularly in the past decade (2013–2023), there has been a significant increase in both the volume of publications and citation counts. This surge may be attributed to the 2013 white paper by the International Association of Human-Animal Interaction Organizations (IAHAIO), titled “The IAHAIO Definitions for Animal Assisted Activity and Guidelines for Wellness of Animals Involved,” which formally defined AAI as a goal-oriented intervention incorporating animals into health, education, and human services to achieve therapeutic benefits for humans (49). This document may have catalyzed global research into the incorporation of animals into health, education, and human services. Subsequent data indicate that the peak in publication numbers in 2020 (n=54) and citation counts in 2021 (n=1,360) further underscores the growing significance of AAI as a therapeutic approach for mental and physical health in recent years.

Analysis of core journals and highly cited research in AAI reveals that the impact factors of the 11 core journals are generally low, with limited publication volumes. Even the journal with the highest number of publications, *Animals*, only has 26 articles. This may reflect that AAI, as an adjunctive therapy, is still in its developmental or relatively niche phase compared to more established medical or psychological interventions, suggesting a need for broader and deeper research coverage. Of the ten most-cited papers, nine are reviews. When review articles are heavily cited within a field, it often indicates rapid development in that area, with researchers summarizing and integrating new knowledge to delineate current research boundaries and future directions. Notably, the most cited article is “Animal-Assisted Intervention for Autism Spectrum Disorder: A Systematic Literature Review” (22). Additionally, four reviews focus on dementia (11, 25, 26, 28), indicating significant therapeutic effects or potential of AAI in treating autism spectrum disorder and dementia, such as increased social interaction among autistic children (22) and improved social behaviors with reduced agitation and aggression in dementia patients (11, 28).

The global distribution of research productivity and impact in the AAI field reflects various factors, including national research investment, academic resources, and the extent of international collaboration. The United States leads in productivity (n=184) and the number of top ten high-producing authors (n=5), which is closely related to its substantial research funding and extensive academic resources. The United States hosts numerous universities and research institutions dedicated to fields such as medicine, psychology, and veterinary science, which are closely linked to AAI, with Purdue University contributing the most in this field. Italy (n=53) and Australia (n=35) rank second and third, respectively, reflecting their active engagement in AAI research. Italy’s long history in medical and psychological research provides a solid theoretical foundation for AAI, and its Ministry of Health has established “National Guidelines for AAI,” standardizing procedures for AAI (50), which may facilitate practical applications. Australia’s high ranking may be attributed to its emphasis on mental health and animal welfare, particularly through its model for animal welfare assessment and its continuous updates (51–57), which likely promotes the widespread application of AAI in practice. The high CPA in Australia (CPA=31.03) and Italy (CPA=27.43) further reflects both countries’ focus on innovative and targeted research directions that have attracted significant international academic attention and citations.

From a cross-national collaboration perspective, the network of collaborations enhances the global progress of AAI research. The United States, positioned at the center of the red cluster, collaborates closely with multiple countries (e.g., China, Canada, the United Kingdom, and Australia), indicating extensive domestic and international research efforts, which elevate the influence and quality of AAI research. European countries (e.g., Spain, Italy, the Netherlands, Switzerland, Germany, and Hungary) also demonstrate close collaborations, reflecting Europe’s emphasis on multidisciplinary research cultures. With the increasing global focus on mental health and overall well-being, AAI research is expected to continue growing. To further enhance the impact and practical value of research, countries should continue to strengthen international collaborations, particularly in data sharing, standardized research methodologies, and cross-cultural studies. Establishing a global AAI research network can enable countries to collectively address global health challenges and advance AAI applications and research across broader social and cultural contexts.

The analysis of high-frequency words reveals the core hotspots and trends in the research field of AAI. Among them, the high frequency of “therapy” and “health” shows that AAI is mainly used to promote physical and mental health, especially in coping with “depression” [e.g., (58, 59)],”stress” [e.g., (60, 61)] and “anxiety” [e.g., (62, 63)] and other psychological problems. In addition, the high-frequency appearance of “children” shows that AAI is widely used in this groups [e.g., (64, 65)], while “pet therapy” and “animal-assisted therapy” reflect different intervention modes. It is worth noting that “dog” and “dogs” occupy an important position, indicating that dogs are the most common animals in AAI [e.g., (66, 67, 68)]. These results show that AAI research is continuing to deepen, paying attention to mental health, special population intervention, and the core role of dogs in treatment, which provides important reference for future research and practice.

The thematic evolution of AAI research from 1983 to 2023 indicates a shift from broad foundational research to targeted studies on specific diseases. Early research established the foundational advantages and applications of AAI, while subsequent research increasingly focused on measuring efficacy, addressing specific health conditions, and improving therapeutic interventions. The field’s continuous development reflects a deepening understanding of AAI’s potential and its applications in meeting various psychological and physical health needs. Future research directions should include improving research methodologies, developing new intervention strategies, and conducting more in-depth studies on different populations and diseases to optimize AAI applications further.

The results put forward some practical application and policy significance. First, governments and medical institutions should consider incorporating AAI into mental health and rehabilitation programs, especially for conditions such as autism spectrum disorder and dementia, where therapeutic effects are well supported. Second, developing standardized protocols and training for practitioners involved in AAI can ensure ethical practices and improve intervention effectiveness. Finally, promoting international collaboration and creating multidisciplinary research alliances could expand AAI’s reach and address cultural differences in its application, paving the way for more comprehensive and impactful interventions.

## 5 Conclusion

To the best of our knowledge, this is the inaugural bibliometric study within the domain of AAI. This research analyzed the sources, countries, institutions, authors, and thematic evolution trends of AAI research publications from 1983 to 2023. The findings indicate that AAI research has garnered extensive attention from scholars over the past decade. The most influential countries, institutions, journals, and authors identified are the United States, Purdue University, *Animals*, and Marguerite E. O’Haire, respectively. The “Animal-Assisted Intervention for Autism Spectrum Disorder: A Systematic Literature Review” is the most cited. The focus on specialized interventions for specific populations is likely to be a research trend in the future.

Overall, this study provides a scientific perspective on AAI research and offers valuable insights for researchers, funding agencies, and policymakers. Highlighting current trends and areas of interest, it underscores the potential for future research directions, particularly in developing targeted interventions and expanding the global application and scope of AAI. Enhancing international collaboration and standardizing research methods could further improve the impact and applicability of AAI research across different cultural contexts.

## 6 Limitation

This study has several limitations. Firstly, it exclusively retrieved literature from the WoSCC, which may introduce bias or omissions in the research. Future studies could expand their database coverage by including additional sources, such as Scopus or Google Scholar, to ensure a more comprehensive representation of the field. Secondly, due to limitations in terminology, document types, time range, and language, our search strategy may not encompass all relevant references, potentially affecting the comprehensiveness of the results. Thirdly, the study did not evaluate the potential protective factors of psychotherapy and counseling interventions, which can usually be used to prevent or alleviate symptoms without prescription. Future investigations could focus on integrating these protective factors into intervention strategies to more fully understand their role in AAI. In addition, exploring emerging technologies such as virtual reality or wearables can provide innovative insights into the application and effectiveness of these interventions.

## Data Availability

The original contributions presented in the study are included in the article. Further inquiries can be directed to the corresponding author.
